# *Vibrio* chromosome-specific families

**DOI:** 10.3389/fmicb.2014.00073

**Published:** 2014-03-18

**Authors:** Oksana Lukjancenko, David W. Ussery

**Affiliations:** ^1^Department of Systems Biology, Center for Biological Sequence Analysis, Technical University of DenmarkLyngby, Denmark; ^2^Comparative Genomics Group, Oak Ridge National Laboratory, Biosciences DivisionOak Ridge, TN, USA

**Keywords:** *Vibrio* pan-genome, chromosome-specific genes, *Vibrio* comparative genomics, *Vibrio* core-genome, comparative genomics

## Abstract

We have compared chromosome-specific genes in a set of 18 finished *Vibrio* genomes, and, in addition, also calculated the pan- and core-genomes from a data set of more than 250 draft *Vibrio* genome sequences. These genomes come from 9 known species and 2 unknown species. Within the finished chromosomes, we find a core set of 1269 encoded protein families for chromosome 1, and a core of 252 encoded protein families for chromosome 2. Many of these core proteins are also found in the draft genomes (although which chromosome they are located on is unknown.) Of the chromosome specific core protein families, 1169 and 153 are uniquely found in chromosomes 1 and 2, respectively. Gene ontology (GO) terms for each of the protein families were determined, and the different sets for each chromosome were compared. A total of 363 different “Molecular Function” GO categories were found for chromosome 1 specific protein families, and these include several broad activities: pyridoxine 5' phosphate synthetase, glucosylceramidase, heme transport, DNA ligase, amino acid binding, and ribosomal components; in contrast, chromosome 2 specific protein families have only 66 Molecular Function GO terms and include many membrane-associated activities, such as ion channels, transmembrane transporters, and electron transport chain proteins. Thus, it appears that whilst there are many “housekeeping systems” encoded in chromosome 1, there are far fewer core functions found in chromosome 2. However, the presence of many membrane-associated encoded proteins in chromosome 2 is surprising.

## Introduction

The *Vibrio* genus represents a large subgroup of *Gamma* subdivision of *Proteobacteria*, which are abundant, fast growers that can be highly variable. These bacteria have the ability to form biofilm on biotic and abiotic surfaces and are ubiquitous in marine and estuarine environments at notably high densities in fish, corals, shrimps, plankton, and mammals (Thompson et al., 2004; Reen et al., 2006; Froelich et al., 2013). Currently, the *Vibrio* genus contains more than 60 different species, although complete genome sequences are available for only 10 of them. Several species are known to be pathogenic for humans, fishes, and marine invertebrates, and are well studied. *V. cholerae* can act as the causative agent of the severe and sometimes lethal disease, cholera, and is probably the most sequenced and clinically important member of *Vibrio* species (Heidelberg et al., 2000; Egan and Waldor, 2003). *V. vulnificus* causes septicemia in wound infections; however, despite its high fatality rate, human infections of *V. vulnificus* are rare (Matsuoka et al., 2013; Tsao et al., 2013). *V. parahaemolyticus* and *V. furnissii* infections may lead to gastroenteritis in humans via consumption of raw seafood (Tanabe et al., 2011; Xiang et al., 2013). Strains of *V. anguillarum* species are life threatening to many economically important fish, including Atlantic salmon, seabass, cod, and rainbow trout (Wiik et al., 1995). *V. fischeri* participates in beneficial symbioses with many marine organisms, especially squids (Verma and Miyashiro, 2013). *V. harveyi* causes luminous vibriosis, which infects prawns, oysters, and lobsters (Yu et al., 2013). Finally, *V. splendidus* is known as an extensive bivalve pathogen (Tanguy et al., 2013).

All known *Vibrios* have two chromosomes; the presence of two chromosomes in *V. cholerae* was first documented in 1998 (Trucksis et al., 1998). Chromosome 1 is usually larger, with a relatively constant size of about 3 million base pairs, encoding around 2700 proteins that represent many essential functions. In contrast, chromosome 2 is smaller, about 1 million base pairs encoding roughly a thousand proteins, and contains a highly variable “super-integron” (Rowe-Magnus et al., 1999). *Vibrio* genomes contain many genomic islands, which can contain functions allowing adaptation to specific environments and, perhaps, can even represent speciation events (Vesth et al., 2010).

The existence of two chromosomes in all *Vibrio* genomes, and variance of chromosome 2, has been the main point of many investigations worldwide and has been the subject of multiple discussions about the purpose and origin of smaller chromosomes. It has been proposed that chromosome 2 originated as a megaplasmid, although later Heidelberg et al. have suggested that it may play an important role in the organism and could help optimize the fast replication rate (Okada et al., 2005; Reen et al., 2006; Kirkup et al., 2010; Dikow and Smith, 2013).

The aim of this study is to compare *Vibrio* chromosome specific genes, as well as the conserved core-genome and pan-genomes, across more than 300 strains of the *Vibrio* genus, both complete and available draft genomes, as well as to focus on distribution of functional proteins and available Gene Ontology information between two chromosomes.

## Materials and methods

### Selection and characteristics of bacterial strains

A set of all publically available *Vibrio* strains was selected for this study and downloaded from the NCBI web pages (July 2012). The initial set included 368 genomes, 18 of them were complete and 350 were retrieved as Illumina raw reads from the NCBI Sequence Read Archive (SRA). Of these, 188 genomes were sequenced using a HiSeq 2000 sequencer and the remaining 162 were sequenced with an Illumina Genome Analyzer II.

**Figure 1:**
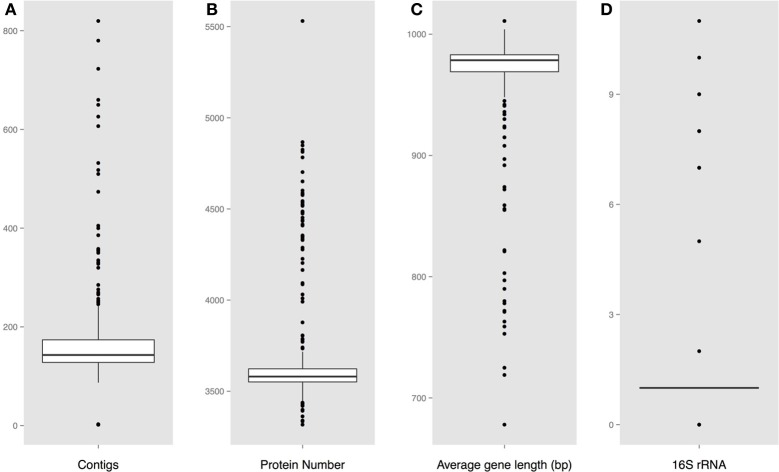
**Predicted genome characteristics **(A)****. Distribution of the number of contiguous pieces **(B)**. Distribution of the protein number per genome **(C)**. Distribution of the average protein coding gene length per genome **(D)**. Number of predicted 16S rRNA sequences.

Protein encoding gene predictions were carried out using the gene-finding tool Prodigal (Hyatt et al., 2010). 16S ribosomal RNA sequences were extracted for both the complete and the draft *Vibrio* genomes using RNAmmer (Lagesen et al., 2007). For each assembled genome, the number of fragments (contiguous pieces), protein coding genes, and the mean gene length were calculated; strains with an average gene length below 700 bp were excluded from further analysis. The resulting set consisted of 18 complete genomes, (Table 1), and 284 draft sequences (Table S1). The distribution of these characteristics for each genome is shown in Figure 1. Note that on average there are about 7 or 8 rRNA operons per complete *Vibrio* genome, although in most draft genomes only one copy is given.

**Table 1:** **List of species used in the study**.

**Strain**	**Chr. I**	**Chr. II**	**Plasmids**
*Vibrio alginolyticus* NBRC 15630^*^	CP006718	CP006719	–
*Vibrio anguillarum* 775	CP002285	CP002284	–
*Vibrio campbellii* ATCC BAA-1116	CP000790	CP000789	CP000791
*Vibrio campbellii* ATCC BAA-1116	CP006606	CP006605	CP006607
*Vibrio cholerae* H1	AKGH01000001	AKGH01000002	–
*Vibrio cholerae* IEC224	CP003331	CP003330	–
*Vibrio cholerae* LMA3984-4	CP002555	CP002556	–
*Vibrio cholerae* M66-2	CP001234	CP001233	–
*Vibrio cholerae* MJ-1236	CP001486	CP001485	–
*V cholerae* O1 El Tor N16961	AE003852	AE003853	–
*Vibrio cholerae* O1 2010EL-1786	CP003070	CP003069	–
*Vibrio cholerae* O395	CP000627	CP000626	–
*Vibrio cholerae* O395	CP001236	CP001235	–
Vibrio furnissii NCTC 11218	CP002377	CP002378	–
*Vibrio nigripulchritudo* SnF1	FO203527	FO203526	–
*Vibrio parahaemolyticus* BB22OP	CP003973	CP003972	–
*V parahaemolyticus* RIMD 2210633	BA000031	BA000032	–
*Vibrio sp*. EJY3	CP003242	CP003241	–
*Vibrio sp*. Ex25	CP001806	CP001805	–
*Vibrio splendidus* LGP32	FM954973	FM954972	–
*Vibrio vulnificus* CMCP6	AE016795	AE016796	–
*Vibrio vulnificus* MO6-24/O	CP002470	CP002469	–
*Vibrio vulnificus* YJ016	BA000037	BA000038	AP005352

* ATCC 17749.

### Proteome comparison

Proteome comparison was performed with the PanFunPro tool (Lukjancenko et al., 2013). Briefly, protein-encoding sequences from each genome were extracted and annotated as described by Lukjancenko et al. (2013) and grouped into protein families. Results of pan- and core-genome analysis for chromosomes 1 and 2 were visualized as an accumulative pan-/core-plot and a pairwise comparison matrix.

The distribution of unique functional profiles between the chromosomes 1 and 2 was examined, followed by a brief investigation of available GO functional categories, specific for each of the chromosomes.

One representative proteome for each species was chosen from the pool of complete genomes and interspecies analysis of specific-genomes was performed between each pair of species. The results were visualized as a specific-matrix.

## Results and discussion

The *Vibrio* dataset consisted of 302 genomes, representing 9 known and 2 unknown *Vibrio* species. A list of the species and accession numbers for the complete genomes is shown in Table 1, and a similar list for all 302 genomes is given in Table S1. Only 18 of the strains were completely finished, and for those independent proteomes for both chromosomes 1 and 2 were extracted. However, most of the genomes (284) were draft and partially assembled into several large pieces of continuous chromosomal DNA, although information concerning which protein belongs to which chromosome was not available. Thus, it was decided to build analysis around 2 sets: the finished genomes (18 genomes) and the whole dataset, including the WGS draft genomes (302 genomes).

The calculated basic features for each analyzed genome is shown in Figure 1, including the number of contiguous pieces, predicted protein coding genes, average gene lengths, and predicted 16S rRNAs. A large fraction of the assembled genomes contain between 150 and 190 contiguous pieces (contigs) of chromosomal DNA, with a group of outlier strains showing more than 200 pieces per genome. An obvious correlation can be seen between the number of contigs and the amount of predicted rRNAs and genes, followed by a shorter than average gene length in assembled genomes with higher numbers of contiguous sequences.

### *Vibrio cholerae* chromosome 1 and chromosome 2 comparison

**Figure 2:**
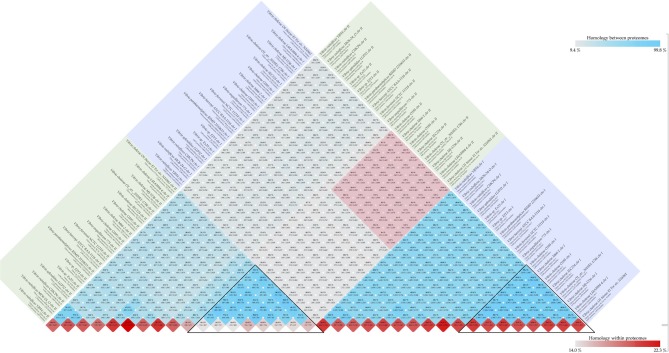
***Vibrio* chromosome comparison**. Comparison was performed for set of 18 genomes. The blue and green square boxes represent chromosomes 1 and 2, respectively. The red-colored box in the middle of the figure indicates inter-chromosomal comparison of *V. cholerae* species, and the black-colored triangles highlight similarities within the same chromosome of the species.

The *Vibrio cholerae* chromosome 1 is larger (about 3 Mbp) and is more stable, carrying many essential protein coding genes, whereas chromosome 2 is smaller (about 1 Mbp), contains a large genomic island (the “superintegron”), is more variable, and has fewer essential genes. A pairwise comparison of set of 18 genomes for both chromosomes is shown in Figure 2. Chromosomes 1 and 2 share a bit more than 10% of their protein families. Within chromosome 1 the range is 55 to 96%, and for chromosome 2 it is 25 to 95%. Since there are multiple genome sequences for several different strains available for the *V. cholerae* species, a high similarity within chromosomes can be found with confidence, although on average only 10% of the proteins are shared between chromosomes 1 and 2.

The core-genome of complete strains contains 1269 conserved protein families shared within chromosome 1, and 252 core families shared within chromosome 2; only 104 functional profiles are shared between the two chromosomes. When additional draft genomes were included, the numbers for both chromosome 1 and 2 dropped to 673 core-genomes and 140 protein families, followed by a decrease of shared functional profiles for a total number of 96. The core- and pan-genome summary results are shown in Table 2 and conserved profiles and their functions in Table S2.

**Table 2:** **List of species analyzed in this study**.

	**18 genomes**	**302 genomes**
**CORE-GENOME**		
Chromosome 1	1269	673
Chromosome 2	252	140
Both chromosomes	104	96
**PAN-GENOME**		
Chromosome 1	5498	NA
Chromosome 2	3742	NA
Both chromosomes	7825	17363

For each species the number of available genomes and sequence status are provided. Species are listed alphabetically.

The pan genome for chromosome 1 (~5500 gene families) is about twice the number of genes encoded in a single copy of chromosome 1 (e.g., 2650 genes in *V. cholerae* strain M66-2), whilst the pan-genome for chromosome 2 (~3740 gene families) is more than three times the size found encoded in a single copy of chromosome 2 (e.g., 1043 genes for *V. cholerae* strain M66-2). Many of these additional gene families are likely to be found in the super-integron, which is a known variable region of chromosome 2.

**Figure 3:**
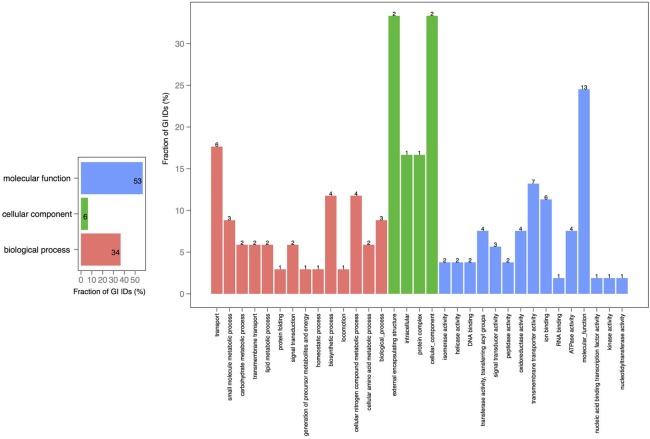
**GO term analysis in proteins shared by chromosomes 1 and 2**. The distribution is shared both as percentage on the axis and the absolute number above the bar. The absolute number reflects the amount of GO IDs that were connected to the pathway. The color code is as follows: red is the biological process, green is the cellular component, and blue is the molecular function.

A closer look at the distribution of functions within the core-genomes of two chromosomes showed that all of the shared proteins are found in the PfamA database (Figure S1) and most of them are involved in biological processes or molecular function (Figure 3). The presence of proteins involved in essential metabolic and regulatory processes in the shared genomic pool of both chromosomes is consistent with the claim that the smaller chromosome is not a plasmid, but is fundamental for growth and biological activity.

**Figure 4:**
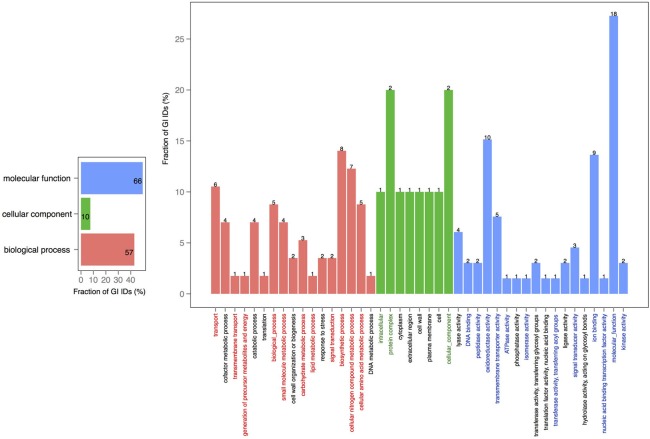
**GO term analysis in protein coding genes shared within chromosome 1 and missing in the core of chromosome 2**. The distribution is shared both as percentage on the axis and the absolute number above the bar. The absolute number shows the amount of GO IDs that were connected to the pathway. The color code is as follows: red is the biological process, green is the cellular component, and blue is the molecular function.

**Figure 5:**
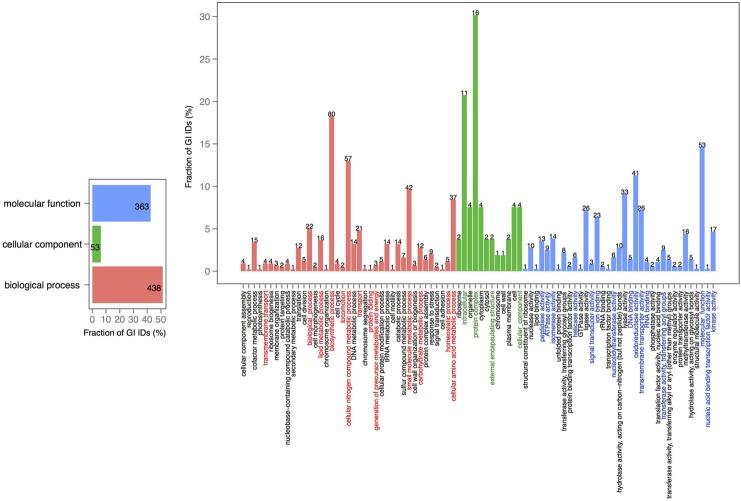
**GO term analysis in protein coding genes shared within chromosome 2 and missing in the core of chromosome 1**. The distribution is shared both as percentage on the axis and the absolute number above the bar. The absolute number shows the amount of GO IDs that were connected to the pathway. The color code is as follows: red is the biological process, green is the cellular component, and blue is the molecular function.

In order to explore the overlap between the core genes in chromosomes 1 and 2, we extracted the core proteins for each chromosome and then examined the overlap with the core of the other chromosome (Figures 4, 5). A total number of 639 GO IDs could be extracted for the chromosome 1 core-specific profiles (1169 profiles). 438 of these were involved in biological processes, 53 in cellular component functions, and 363 in molecular functions. Equivalent analysis of chromosome 2 core-specific profiles yielded, in total, 109 GO IDs (of 153 profiles). 57 of the IDs were involved in biological processes, 10 in cellular components, and 66 in molecular functions. It is not surprising that whilst the core of chromosome 1 carries more proteins that are essential to sustain life and to reproduce, the specific core of chromosome 2 contains proteins involved in metabolic processes and enzyme and membrane associated activity. The addition of 284 draft genomes slightly reduced the number of specific proteins and specific pathway groups in chromosome 1, leaving 265 GO terms involved in the biological process, 39 in cellular component functions, and 197 in molecular functions (Figure S2). In contrast, chromosome 2 contained 15 GO terms in biological processes, 4 in cellular components, and 14 in molecular functions (Figure S3).

### Species comparison

**Figure 6:**
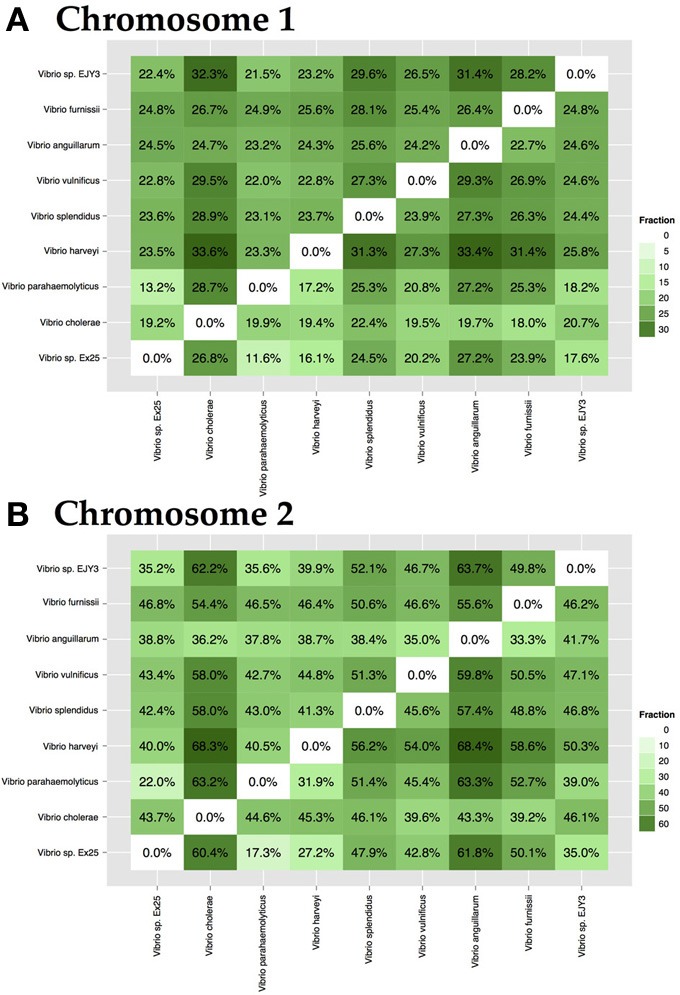
**Pairwise interspecies-specific genome comparison for chromosome 1 (A) and chromosome 2 (B)**. Analysis included a single representation of 7 known and 2 unknown species. The resulting percentage shows the ratio between the amount of species-specific families and the size of the total proteome. On average, each species contained between 18 and 33% specific protein families. Color intensity indicates the level of specificity.

The genus *Vibrio* is comprised of a diverse group of bacteria, which can be either pathogenic or symbiotic to mammals and organisms of marine environments. Species-specific genomes may contain proteins responsible for pathogenicity or they may be crucial for survival in a given environment. To demonstrate the level of specificity between species of the same chromosome, 9 strains representing 7 known and 2 unknown species, a pairwise comparison of specific-genomes, was performed. Within chromosome 1, the fraction of unique proteomes varies from 18 to 33% (Figure 6A), whereas genomes of chromosome 2 differ in a greater portion of proteins, ranging from 18 to 64% (Figure 6B).

*Vibrio cholerae* spp. are known pathogens in humans and were chosen to examine for genome specific differences in gene content. Representative strains of *V. cholerae* species were compared to other strains, as shown in Figure S4. Chromosomes 1 and 2 contained a similar amount of specific profiles, 190, and 192, respectively. Most of them were CD-HIT clustering-based, however, 79 and 44 were annotated by PfamA and TIGRFAM collections. A complete list of profiles and corresponding functions are listed in Table S3.

### Proteomes of *V. cholerae* draft genomes

**Figure 7:**
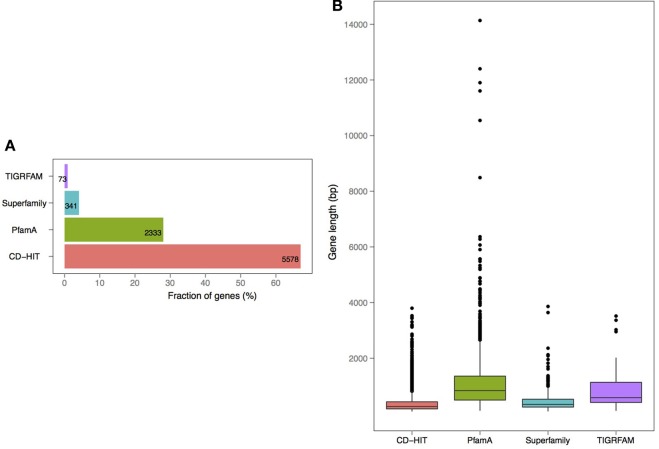
**Annotation and length distribution of proteins within specific-proteomes in draft genomes of *V. cholerae* (A)**. Distribution of profiles by assignment source: PfamA, Superfamily, TIGRFAM, and CD-HIT clustering **(B)**. Protein coding gene length distribution by each profile type.

**Figure 8:**
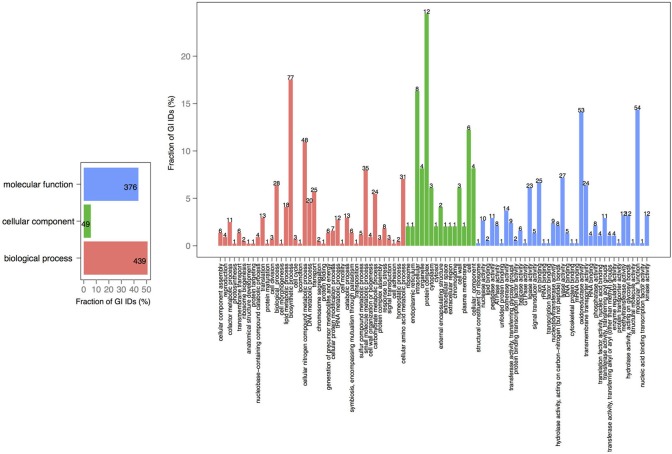
**GO term analysis in proteins, specific to *V. cholerae* draft genomes**. Distribution is shared both as the percentage on the axis and the absolute number above the bar. The absolute number shows the amount of GO IDs that were connected to the pathway. The color code is as follows: red is the biological process, green is the cellular component, and blue is the molecular function.

*V. cholerae* is one of the most important, highly documented, and most sequenced species of *Vibrios*. Our dataset included 279 *V. cholerae* strains, 8 completely sequenced and 271 draft genomes. For the draft genomes, chromosome specific genes could not be calculated. However, starting with the known core genomes from the finished genomes, it is possible to look for the presence of the known chromosome core genes across the draft genomes. Thus, core-genome analysis of 279 *V. cholerae* strains yielded in 776, 250, and 182 protein families, in large, small, and both of the chromosomes, respectively. Further, we examined the pan-genomes of both chromosomes within a set of 18 genomes. The distribution of the total number of 8325 functional profiles is as follows: 2333, 341 and 73 families assigned to PfamA, Superfamily, and TIGRFAM databases, respectively (Figure 7). We estimate that the 271 newly sequenced *V. cholerae* strains brings at least 2000 possible profile combinations to the pool of previously known functions that represent more than 70 different GO functional categories (Figure 8).

In conclusion, the *Vibrio* pan-genome can be quite large, with more than 17,000 gene families, although, any one *Vibrio* genome will contain only about 3500 genes, or about one-fifth of the size of the pan-genome. There is considerably more variability in chromosome 2 than in chromosome 1.

### Conflict of interest statement

The authors declare that the research was conducted in the absence of any commercial or financial relationships that could be construed as a potential conflict of interest.

## Supplementary material

Figure S1 **Annotation and length distribution of proteins within core-genome of small and large chromosomes (A)**. Distribution of profiles by assignment source: PfamA, Superfamily, TIGRFAM, and CD-HIT clustering **(B)**. Protein coding gene length distribution by each profile type.

Figure S2 **GO term analysis in proteins shared within chromosome 1 and missing in the core of chromosome 2 in set_302 genomes**. Distribution is shared both as percentage on the axis and absolute number above the bar. Absolute number shows the amount of GO IDs that were connected to the pathway. Color code is as follows: red is biological process, green is cellular component, and blue is molecular function.

Figure S3 **GO term analysis in proteins shared within chromosome 2 and missing in the core of chromosome 1 in set_302 genomes**. Distribution is shared both as percentage on the axis and absolute number above the bar. Absolute number shows the amount of GO IDs that were connected to the pathway. Color code is as follows: red is biological process, green is cellular component, and blue is molecular function.

Figure S4 **Annotation and length distribution of proteins within *V. cholerae* species-specific proteomes for chromosome 1 **(panel A)** and chromosome 2 **(panel B)****. Annotation of profiles and protein coding gene length distribution are visualized by assignment source: PfamA, Superfamily, TIGRFAM, and CD-HIT clustering.

Table S1 **List of Sequence Read Archive (SRA) genomes used in the study**.

Table S2 **Conserved functional profiles within genomes of set_18**. Whether profile consists of more than 1 domain, function is shown for each domain.

Table S3 **Profiles, specific for *V. cholerae* species., in chromosome I and chromosome II**. Whether profile consists of more than 1 domain, function is shown for each domain.
