# Supplementary material for: Vibrio chromosome-specific families
Source: Front Microbiol. 2014 Mar 18;5:73. doi: 10.3389/fmicb.2014.00073 (PMC3957060; doi:10.3389/fmicb.2014.00073)
Supplement: Figure S1 — Annotation and length distribution of proteins within core-genome of small and large chromosomes (A). Distribution of profiles by assignment source: PfamA, Superfamily, TIGRFAM, and CD-HIT clustering (B). Protein coding gene length distribution by each profile type. [file DataSheet1.PDF]

Supplemental Data

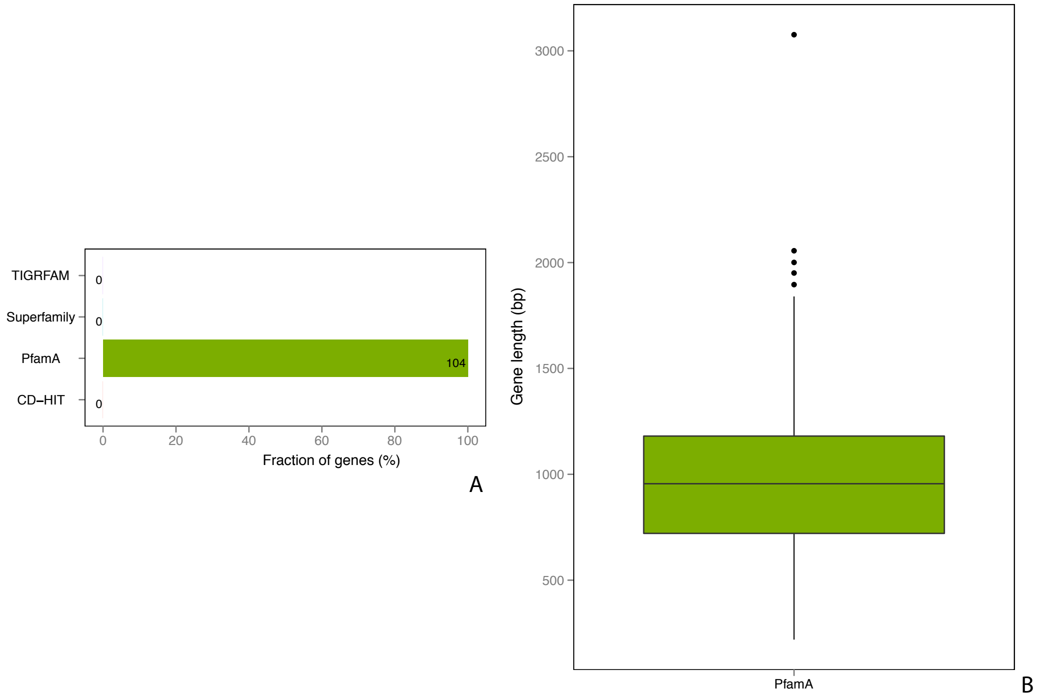

Figure S1: Annotation and length distribution of proteins within core-genome of small and large chromosomes. A. Distribution of profiles by assignment source: PfamA, Superfamily, TIGRFAM, and CD-HIT clustering. B. Protein coding gene length distribution by each profile type.

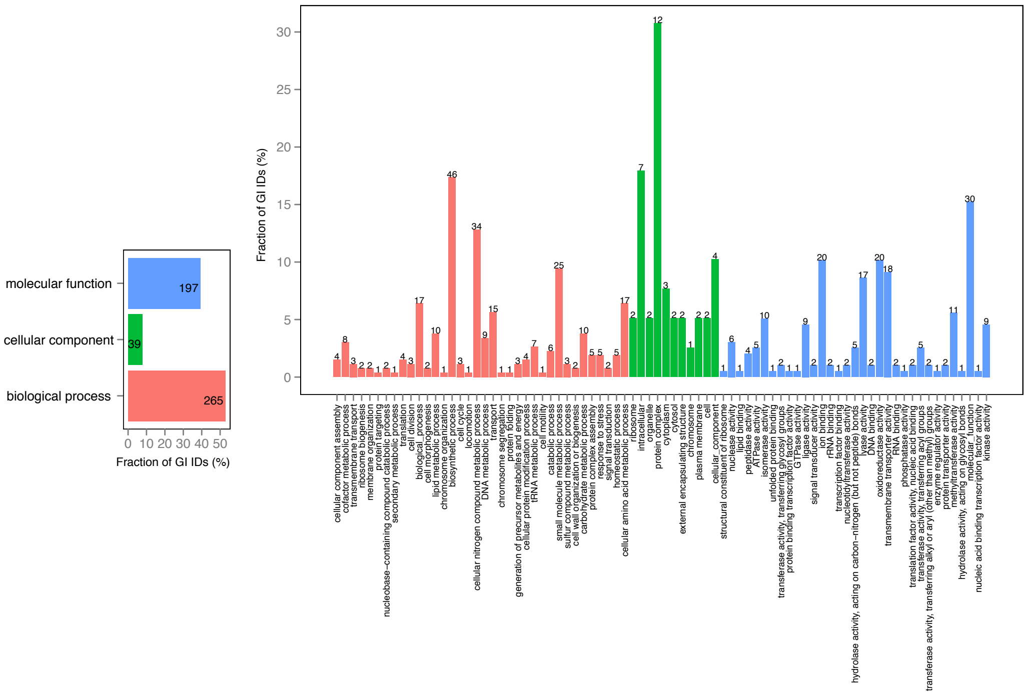

Figure S2: GO term analysis in proteins shared within chromosome 1 and missing in the core of chromosome 2 in set_302 genomes. Distribution is shared both as percentage on the axis and absolute number above the bar. Absolute number shows the amount of GO IDs that were connected to the pathway. Colour code is as follows: red is biological process, green is cellular component, and blue is molecular function.

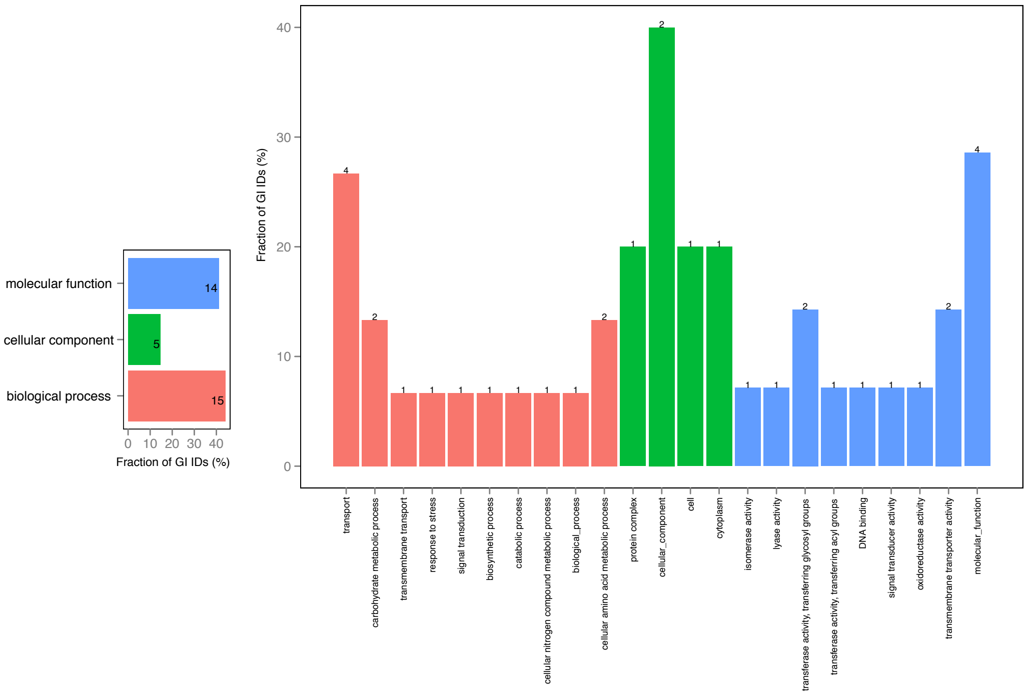

Figure S3: GO term analysis in proteins shared within chromosome 2 and missing in the core of chromosome 1 in set_302 genomes. Distribution is shared both as percentage on the axis and absolute number above the bar. Absolute number shows the amount of GO IDs that were connected to the pathway. Colour code is as follows: red is biological process, green is cellular component, and blue is molecular function.

Table S1. List of Sequence Read Archive (SRA) genomes used in the study.

Organism	Accession	N50
Vibrio cholerae BJG-01	SRR135539	144947
Vibrio cholerae CP1030 3	SRR227318	259494
Vibrio cholerae CP1032 5	SRR227312	214291
Vibrio cholerae CP1037 10	SRR227319	178804
Vibrio cholerae CP1038 11	SRR227311	169047
Vibrio cholerae CP1040 13	SRR227307	151808
Vibrio cholerae CP1041 14	SRR227309	152490
Vibrio cholerae CP1042 15	SRR227324	246222
Vibrio cholerae CP1044 17	SRR227336	344367
Vibrio cholerae CP1046 19	SRR227322	230105
Vibrio cholerae CP1047 20	SRR227334	251440
Vibrio cholerae CP1048 21	SRR227303	165981
Vibrio cholerae CP1050 23	SRR227335	235725
Vibrio cholerae	ERR018111	178092
Vibrio cholerae	ERR018112	152665
Vibrio cholerae	ERR018113	43637
Vibrio cholerae	ERR018114	152819
Vibrio cholerae	ERR018115	108927
Vibrio cholerae	ERR018116	35936
Vibrio cholerae	ERR018117	13439
Vibrio cholerae	ERR018123	202944
Vibrio cholerae	ERR018125	52477
Vibrio cholerae	ERR018138	168906
Vibrio cholerae	ERR018139	163190
Vibrio cholerae	ERR018145	200960
Vibrio cholerae	ERR018146	250757
Vibrio cholerae	ERR018148	204502
Vibrio cholerae	ERR018149	468918
Vibrio cholerae	ERR018150	259322
Vibrio cholerae	ERR018151	288215
Vibrio cholerae	ERR018152	259834
Vibrio cholerae	ERR018153	125249
Vibrio cholerae	ERR018154	246976
Vibrio cholerae	ERR018155	202271
Vibrio cholerae	ERR018156	327064
Vibrio cholerae	ERR018158	203673
Vibrio cholerae	ERR018164	282089
Vibrio cholerae	ERR018165	155521
Vibrio cholerae	ERR018184	199423
Vibrio cholerae	ERR018185	54277
Vibrio cholerae	ERR018187	230150
Vibrio cholerae	ERR018188	218446
Vibrio cholerae	ERR018189	227365
Vibrio cholerae	ERR018191	246851
Vibrio cholerae	ERR018192	98591
Vibrio cholerae	ERR018193	40321

Vibrio cholerae	ERR018195	198376
Vibrio cholerae	ERR019879	102287
Vibrio cholerae	ERR019880	75585
Vibrio cholerae	ERR019881	75831
Vibrio cholerae	ERR019882	75539
Vibrio cholerae	ERR019883	35793
Vibrio cholerae	ERR019884	69675
Vibrio cholerae	ERR019885	21257
Vibrio cholerae	ERR025356	536569
Vibrio cholerae	ERR025357	202406
Vibrio cholerae	ERR025358	536582
Vibrio cholerae	ERR025359	299286
Vibrio cholerae	ERR025360	235682
Vibrio cholerae	ERR025361	246247
Vibrio cholerae	ERR025362	355189
Vibrio cholerae	ERR025363	218510
Vibrio cholerae	ERR025364	737499
Vibrio cholerae	ERR025366	536722
Vibrio cholerae	ERR025367	450450
Vibrio cholerae	ERR025368	299130
Vibrio cholerae	ERR025369	246232
Vibrio cholerae	ERR025370	299511
Vibrio cholerae	ERR025371	246198
Vibrio cholerae	ERR025372	319384
Vibrio cholerae	ERR025373	246412
Vibrio cholerae	ERR025374	299381
Vibrio cholerae	ERR025375	260907
Vibrio cholerae	ERR025377	555162
Vibrio cholerae	ERR025378	456380
Vibrio cholerae	ERR025379	536743
Vibrio cholerae	ERR025380	252984
Vibrio cholerae	ERR025381	456170
Vibrio cholerae	ERR025382	537064
Vibrio cholerae	ERR025383	296837
Vibrio cholerae	ERR025384	259485
Vibrio cholerae	ERR025385	537284
Vibrio cholerae	ERR025386	355549
Vibrio cholerae	ERR025388	456004
Vibrio cholerae	ERR025389	356078
Vibrio cholerae	ERR025390	394321
Vibrio cholerae	ERR025391	238402
Vibrio cholerae	ERR025392	356379
Vibrio cholerae	ERR025393	246394
Vibrio cholerae	ERR025394	365595
Vibrio cholerae	ERR025395	246218
Vibrio cholerae	ERR025396	519994
Vibrio cholerae	ERR028066	163546
Vibrio cholerae	ERR028068	166412
Vibrio cholerae	ERR028074	235768

Vibrio cholerae	ERR028075	196904
Vibrio cholerae	ERR028076	134862
Vibrio cholerae	ERR037705	246344
Vibrio cholerae	ERR037706	246364
Vibrio cholerae	ERR037707	125408
Vibrio cholerae	ERR037708	200249
Vibrio cholerae	ERR037709	221796
Vibrio cholerae	ERR037710	133654
Vibrio cholerae	ERR037711	200261
Vibrio cholerae	ERR037712	349589
Vibrio cholerae	ERR037713	253384
Vibrio cholerae	ERR037714	200428
Vibrio cholerae	ERR037715	355897
Vibrio cholerae	ERR037716	246506
Vibrio cholerae	ERR037717	237467
Vibrio cholerae	ERR037718	299402
Vibrio cholerae	ERR037719	299433
Vibrio cholerae	ERR037720	299410
Vibrio cholerae	ERR037721	246401
Vibrio cholerae	ERR037722	299420
Vibrio cholerae	ERR037723	246073
Vibrio cholerae	ERR037724	299458
Vibrio cholerae	ERR037725	246433
Vibrio cholerae	ERR037726	246445
Vibrio cholerae	ERR037727	299418
Vibrio cholerae	ERR037728	537444
Vibrio cholerae	ERR037729	246297
Vibrio cholerae	ERR037730	739245
Vibrio cholerae	ERR037731	345556
Vibrio cholerae	ERR037732	299482
Vibrio cholerae	ERR037733	246423
Vibrio cholerae	ERR037734	246407
Vibrio cholerae	ERR037735	319677
Vibrio cholerae	ERR037736	246437
Vibrio cholerae	ERR037737	61985
Vibrio cholerae	ERR037738	299407
Vibrio cholerae	ERR037739	303139
Vibrio cholerae	ERR037740	246480
Vibrio cholerae	ERR037741	303002
Vibrio cholerae	ERR037742	65519
Vibrio cholerae	ERR037743	78515
Vibrio cholerae	ERR037744	80282
Vibrio cholerae	ERR037745	246297
Vibrio cholerae	ERR037746	345653
Vibrio cholerae	ERR037747	555651
Vibrio cholerae	ERR037748	299563
Vibrio cholerae	ERR037749	299417
Vibrio cholerae	ERR037750	198990
Vibrio cholerae	ERR039258	246344

Vibrio cholerae	ERR039259	235743
Vibrio cholerae	ERR039260	199271
Vibrio cholerae	ERR039261	199357
Vibrio cholerae	ERR039262	200211
Vibrio cholerae	ERR039263	299156
Vibrio cholerae	ERR039264	299293
Vibrio cholerae	ERR039265	299241
Vibrio cholerae	ERR039266	299320
Vibrio cholerae	ERR039267	299139
Vibrio cholerae	ERR039268	345428
Vibrio cholerae	ERR039269	199356
Vibrio cholerae	ERR039270	203979
Vibrio cholerae	ERR039271	299267
Vibrio cholerae	ERR039272	246271
Vibrio cholerae	ERR039273	299254
Vibrio cholerae	ERR039274	345470
Vibrio cholerae	ERR039275	246349
Vibrio cholerae	ERR039276	299335
Vibrio cholerae	ERR039277	345408
Vibrio cholerae	ERR039278	299350
Vibrio cholerae	ERR039279	246303
Vibrio cholerae	ERR039280	197860
Vibrio cholerae	ERR039281	299137
Vibrio cholerae	ERR039282	346029
Vibrio cholerae	ERR039283	235777
Vibrio cholerae	ERR039284	235747
Vibrio cholerae	ERR039285	204043
Vibrio cholerae	ERR039286	197796
Vibrio cholerae	ERR039287	232165
Vibrio cholerae	ERR039288	232130
Vibrio cholerae	ERR039289	232187
Vibrio cholerae	ERR039290	232229
Vibrio cholerae	ERR039291	232214
Vibrio cholerae	ERR039292	232099
Vibrio cholerae	ERR039293	232197
Vibrio cholerae	ERR039294	197740
Vibrio cholerae	ERR039295	235711
Vibrio cholerae	ERR039296	232198
Vibrio cholerae	ERR039297	204026
Vibrio cholerae	ERR039298	197816
Vibrio cholerae	ERR039299	232190
Vibrio cholerae	ERR039300	232140
Vibrio cholerae	ERR039301	235799
Vibrio cholerae	ERR039302	235688
Vibrio cholerae	ERR039303	158486
Vibrio cholerae	ERR039304	197806
Vibrio cholerae	ERR039305	136194
Vibrio cholerae	ERR039306	137037
Vibrio cholerae	ERR039307	197758

Vibrio cholerae	ERR039308	199372
Vibrio cholerae	ERR039309	199360
Vibrio cholerae	ERR039310	299351
Vibrio cholerae	ERR039311	299328
Vibrio cholerae	ERR039312	258004
Vibrio cholerae	ERR039313	345415
Vibrio cholerae	ERR039314	345709
Vibrio cholerae	ERR039315	299430
Vibrio cholerae	ERR039316	246350
Vibrio cholerae	ERR039317	325213
Vibrio cholerae	ERR039318	299295
Vibrio cholerae	ERR039319	299376
Vibrio cholerae	ERR039320	345481
Vibrio cholerae	ERR039321	299305
Vibrio cholerae	ERR039322	299369
Vibrio cholerae HC-02A1	SRR135602	179570
Vibrio cholerae HC-06A1	SRR190870	246259
Vibrio cholerae HC-17A1	SRR346409	199338
Vibrio cholerae HC-19A1	SRR191384	246244
Vibrio cholerae HC-1A2	SRR135604	113635
Vibrio cholerae HC-20A2	SRR191382	197792
Vibrio cholerae HC-20A2	SRR233066	197784
Vibrio cholerae HC-21A1	SRR191389	299442
Vibrio cholerae HC-22A1	SRR191381	338465
Vibrio cholerae HC-23A1	SRR135546	176574
Vibrio cholerae HC-28A1	SRR191380	235957
Vibrio cholerae HC-32A1	SRR191391	356657
Vibrio cholerae HC-33A2	SRR191386	299348
Vibrio cholerae HC-36A1	SRR191346	190278
Vibrio cholerae HC-38A1	SRR135605	199330
Vibrio cholerae HC-39A1	SRR227325	246734
Vibrio cholerae HC-40A1	SRR135545	134457
Vibrio cholerae HC-41A1	SRR227304	197849
Vibrio cholerae HC-41B1	SRR350020	162602
Vibrio cholerae HC-42A1	SRR227326	198024
Vibrio cholerae HC-43A1	SRR135603	30532
Vibrio cholerae HC-43B1	SRR212959	32320
Vibrio cholerae HC-46A1	SRR221648	246580
Vibrio cholerae HC-47A1	SRR227327	246518
Vibrio cholerae HC-48A1	SRR135621	130601
Vibrio cholerae HC-48B2	SRR191383	143184
Vibrio cholerae HC-49A2	SRR135544	199330
Vibrio cholerae HC-50A1	SRR227305	185434
Vibrio cholerae HC-51A1	SRR227330	153200
Vibrio cholerae HC-52A1	SRR227331	126869
Vibrio cholerae HC-55A1	SRR227332	185606
Vibrio cholerae HC-55C2	SRR346408	185288
Vibrio cholerae HC-56A1	SRR227328	179884
Vibrio cholerae HC-56A2	SRR227329	230301

Vibrio cholerae HC-57A1	SRR227316	153994
Vibrio cholerae HC-57A2	SRR227306	197785
Vibrio cholerae HC-59A1	SRR341227	124707
Vibrio cholerae HC-61A1	SRR135543	25049
Vibrio cholerae HC-61A2	SRR346406	153877
Vibrio cholerae HC-62A1	SRR346411	199331
Vibrio cholerae HC-64A1	SRR191347	246266
Vibrio cholerae HC-65A1	SRR190877	246335
Vibrio cholerae HC-67A1	SRR191351	246333
Vibrio cholerae HC-68A1	SRR191363	246378
Vibrio cholerae HC-70A1	SRR135607	166665
Vibrio cholerae HC-71A1	SRR191349	299581
Vibrio cholerae HC-72A2	SRR191719	218493
Vibrio cholerae HC-78A1	SRR190873	151294
Vibrio cholerae HC-7A1	SRR191348	198662
Vibrio cholerae HC-80A1	SRR191350	166883
Vibrio cholerae HC-81A1	SRR191343	235786
Vibrio cholerae HC-81A2	SRR227333	236336
Vibrio cholerae HCUF01	SRR135540	198616
Vibrio cholerae HE-09	SRR135541	196941
Vibrio cholerae HE-16	SRR227317	329739
Vibrio cholerae HE-45	SRR221551	237060
Vibrio cholerae HE-46	SRR346405	142246
Vibrio cholerae HE39	SRR135547	79441
Vibrio cholerae HE48	SRR135542	185904
Vibrio cholerae HFU-02	SRR135620	246201
Vibrio cholerae O1 str. 2010EL-1786	SRR073556	14022
Vibrio cholerae O1 str. 2010EL-1792	SRR074112	15216
Vibrio cholerae O1 str. 2010EL-1798	SRR074109	14191
Vibrio alginolyticus 12G01	SRR022567	13877
Vibrio fischeri ES114	SRR072792	277223
Vibrio splendidus	SRR022569	33819
Vibrio splendidus	SRR022570	39534
Vibrio splendidus	SRR022571	33709
Vibrio splendidus	SRR022573	38847
Vibrio splendidus	SRR022575	71292
Vibrio splendidus	SRR022576	74787
Vibrio splendidus	SRR022577	51882
Vibrio splendidus	SRR022578	75732
Vibrio splendidus	SRR022579	54802
Vibrio splendidus	SRR022580	66533
Vibrio splendidus	SRR022581	59776

Table S2. Conserved functional profiles within genomes of set_18. Whether profile consists of more than 1 domain, function is shown for each domain.

Functional Profile	Function
PF00497	- Bacterial extracellular solute-binding proteins family 3
PF01554	- MatE
PF00512_PF00672_PF02518	- His Kinase A (phospho-acceptor) domain<br>- HAMP domain<br>- Histidine kinase- DNA gyrase B- and HSP90-like ATPase
PF00072_PF01584	- Response regulator receiver domain<br>- CheW-like domain
PF13416	- Bacterial extracellular solute-binding protein
PF13302	- Acetyltransferase (GNAT) domain
PF00072	- Response regulator receiver domain
PF00072_PF00196	- Response regulator receiver domain<br>- Bacterial regulatory proteins luxR family
PF01979	- Amidohydrolase family
PF01546_PF07687	- Peptidase family M20/M25/M40<br>- Peptidase dimerisation domain
PF01323	- DSBA-like thioredoxin domain
PF00383	- Cytidine and deoxycytidylate deaminase zinc-binding region
PF00581	- Rhodanese-like domain
PF01553	- Acyltransferase
PF00462	- Glutaredoxin
PF00899_PF05237	- ThiF family||MoeZ/MoeB domain
PF02311_PF12833	- AraC-like ligand binding domain<br>- Helix-turn-helix domain
PF13343	- Bacterial extracellular solute-binding protein
PF01467	- Cytidylyltransferase
PF02355_PF07549_PF13721	- Protein export membrane protein<br>- SecD/SecF GG Motif<br>- SecD export protein N-terminal TM region
PF00126_PF03466	- Bacterial regulatory helix-turn-helix protein lysR family<br>- LysR substrate binding domain
PF00392_PF07702	- Bacterial regulatory proteins gntR family<br>- UTRA domain
PF00753	- Metallo-beta-lactamase superfamily
PF00464	- Serine hydroxymethyltransferase
PF00155	- Aminotransferase class I and II
PF00072_PF00486	- Response regulator receiver domain<br>- Transcriptional regulatory protein C terminal
PF03009	- Glycerophosphoryl diester phosphodiesterase family
PF12849	- PBP superfamily domain
PF01032	- FecCD transport family
PF12833	- Helix-turn-helix domain
PF00202	- Aminotransferase class-III
PF00440	- Bacterial regulatory proteins tetR family
PF01926	- 50S ribosome-binding GTPase
PF00483	- Nucleotidyl transferase
PF01545	- Cation efflux family
PF03264	- NapC/NirT cytochrome c family N-terminal region
PF03595	- C4-dicarboxylate transporter/malic acid transport protein

PF01048	- Phosphorylase superfamily
PF01047	- MarR family
PF00583	- Acetyltransferase (GNAT) family
PF01497	- Periplasmic binding protein
PF00005_PF08352	- ABC transporter<br>- Oligopeptide/dipeptide transporter C-terminal region
PF00294	- pfkB family carbohydrate kinase
PF07690	- Major Facilitator Superfamily
PF00496	- Bacterial extracellular solute-binding proteins family 5 Middle
PF00528	- Binding-protein-dependent transport system inner membrane component
PF01810	- LysE type translocator
PF00849	- RNA pseudouridylate synthase
PF00015_PF00672	- Methyl-accepting chemotaxis protein (MCP) signalling domain<br>- HAMP domain
PF00171	- Aldehyde dehydrogenase family
PF08282	- haloacid dehalogenase-like hydrolase
PF00015_PF00672_PF02743	- Methyl-accepting chemotaxis protein (MCP) signalling domain<br>- HAMP domain<br>- Cache domain
PF00278_PF02784	- Pyridoxal-dependent decarboxylase C-terminal sheet domain||Pyridoxal-dependent decarboxylase pyridoxal binding domain
PF00924	- Mechanosensitive ion channel
PF00356_PF13377	- Bacterial regulatory proteins lacI family<br>- Periplasmic binding protein-like domain
PF05618	- Putative ATP-dependant zinc protease
PF00005_PF08402	- ABC transporter<br>- TOBE domain
PF00691	- OmpA family
PF00313	- Cold-shock' DNA-binding domain
PF13508	- Acetyltransferase (GNAT) domain
PF00455_PF08220	- DeoR C terminal sensor domain<br>- DeoR-like helix-turn-helix domain
PF00270_PF00271	- DEAD/DEAH box helicase<br>- Helicase conserved C-terminal domain
PF00375	- Sodium:dicarboxylate symporter family
PF00033	- Cytochrome b(N-terminal)/b6/petB
PF02195	- ParB-like nuclease domain
PF05362_PF13654	- Lon protease (S16) C-terminal proteolytic domain<br>- AAA domain
PF08541_PF08545	- 3-Oxoacyl-[acyl-carrier-protein (ACP)] synthase III C terminal<br>- 3-Oxoacyl-[acyl-carrier-protein (ACP)] synthase III
PF00408_PF02878_PF02879_PF02880	- Phosphoglucomutase/phosphomannomutase C-terminal domain<br>- Phosphoglucomutase/phosphomannomutase alpha/beta/alpha domain I<br>- Phosphoglucomutase/phosphomannomutase alpha/beta/alpha domain II<br>- Phosphoglucomutase/phosphomannomutase alpha/beta/alpha domain III
PF00044_PF02800	- Glyceraldehyde 3-phosphate dehydrogenase NAD binding domain

	- Glyceraldehyde 3-phosphate dehydrogenase C-terminal domain
PF02698	- DUF218 domain
PF03222	- Tryptophan/tyrosine permease family
PF00107_PF08240	- Zinc-binding dehydrogenase<br>- Alcohol dehydrogenase GroES-like domain
PF01925	- Sulfite exporter TauE/SafE
PF00389_PF02826	- D-isomer specific 2-hydroxyacid dehydrogenase catalytic domain<br>- D-isomer specific 2-hydroxyacid dehydrogenase NAD binding domain
PF02811	- PHP domain
PF13419	- Haloacid dehalogenase-like hydrolase
PF00873	- AcrB/AcrD/AcrF family
PF00892	- EamA-like transporter family
PF00903	- Glyoxalase/Bleomycin resistance protein/Dioxygenase superfamily
PF00563_PF00990	- EAL domain<br>- GGDEF domain
PF00293	- NUDIX domain
PF00561	- alpha/beta hydrolase fold
PF03061	- Thioesterase superfamily
PF00465	- Iron-containing alcohol dehydrogenase
PF08238	- Sel1 repeat
PF00254_PF01346	- FKBP-type peptidyl-prolyl cis-trans isomerase<br>- Domain amino terminal to FKBP-type peptidyl-prolyl isomerase
PF13609	- Gram-negative porin
PF01266	- FAD dependent oxidoreductase
PF00005_PF00664	- ABC transporter<br>- ABC transporter transmembrane region
PF00593_PF07715	- TonB dependent receptor<br>- TonB-dependent Receptor Plug Domain
PF01037_PF13412	- AsnC family<br>- Winged helix-turn-helix DNA-binding
PF01569	- PAP2 superfamily
PF12700	- HlyD family secretion protein
PF00106	- short chain dehydrogenase
PF00196	- Bacterial regulatory proteins luxR family
PF00085	- Thioredoxin
PF03605	- Anaerobic c4-dicarboxylate membrane transporter
PF05036	- Sporulation related domain
PF00793	- DAHP synthetase I family
PF00990	- GGDEF domain
PF01656	- CobQ/CobB/MinD/ParA nucleotide binding domain
PF00128	- Alpha amylase catalytic domain
PF00474	- Sodium:solute symporter family
PF00005	- ABC transporter

Table S3. Profiles, specific for *V. cholerae* species., in chromosome I and chromosome II. Whether profile consists of more than 1 domain, function is shown for each domain.

**Functional profile**	**Function**
Chromosome I	
PF00015_PF08447	- Methyl-accepting chemotaxis protein (MCP) signalling domain<br>- PAS fold
PF00028_PF00353	- Cadherin domain<br>- Hemolysin-type calcium-binding repeat (2 copies)
PF00030	- Beta/Gamma crystallin
PF00041_PF10462_PF12561	- Fibronectin type III domain<br>- Peptidase M66<br>- ToxR activated gene A lipoprotein
PF00072_PF08668	- Response regulator receiver domain<br>- HDOD domain
PF00080	- Copper/zinc superoxide dismutase (SODC)
PF00109	- Beta-ketoacyl synthase N-terminal domain
PF00128_PF11852	- Alpha amylase catalytic domain<br>- Domain of unknown function (DUF3372)
PF00142_PF10609	- 4Fe-4S iron sulfur cluster binding proteins NifH/frxC family<br>- ParA/MinD ATPase like
PF00165	- Bacterial regulatory helix-turn-helix proteins AraC family
PF00174	- Oxidoreductase molybdopterin binding domain
PF00199_PF06628	- Catalase||Catalase-related immune-responsive
PF00271_PF02151_PF12344	- Helicase conserved C-terminal domain<br>- UvrB/uvrC motif<br>- Ultra-violet resistance protein B
PF00359_PF00381	- Phosphoenolpyruvate-dependent sugar phosphotransferase system EIIA 2<br>- PTS HPr component phosphorylation site
PF00364_PF02436	- Biotin-requiring enzyme<br>- Conserved carboxylase domain
PF00501_PF00550_PF00668	- AMP-binding enzyme<br>- Phosphopantetheine attachment site<br>- Condensation domain
PF00512_PF02518_PF08448	- His Kinase A (phospho-acceptor) domain<br>- Histidine kinase- DNA gyrase B- and HSP90-like ATPase<br>- PAS fold
PF00515	- Tetratricopeptide repeat
PF00515_PF00990_PF07719_PF13181_PF13424	- Tetratricopeptide repeat<br>- GGDEF domain<br>- Tetratricopeptide repeat<br>- Tetratricopeptide repeat<br>- Tetratricopeptide repeat
PF00571_PF01769	- CBS domain<br>- Divalent cation transporter
PF00593	- TonB dependent receptor
PF00672_PF01966	- HAMP domain<br>- HD domain
PF00672_PF02518	- HAMP domain<br>- Histidine kinase- DNA gyrase B- and HSP90-like ATPase

PF00682_PF02436	- HMGL-like<br>- Conserved carboxylase domain
PF00704_PF02839_PF06483	- Glycosyl hydrolases family 18<br>- Carbohydrate binding domain<br>- Chitinase C
PF00899_PF14461	- ThiF family<br>- Prokaryotic E2 family B
PF00912	- Transglycosylase
PF00919_PF04055	- Uncharacterized protein family UPF0004<br>- Radical SAM superfamily
PF00990_PF07494_PF07495	- GGDEF domain<br>- Two component regulator propeller<br>- Y Y Y domain
PF01344	- Kelch motif
PF01375	- Heat-labile enterotoxin alpha chain
PF01376	- Heat-labile enterotoxin beta chain
PF01490	- Transmembrane amino acid transporter protein
PF01551_PF04225	- Peptidase family M23<br>- Opacity-associated protein A LysM-like domain
PF01752_PF04151_PF08453	- Collagenase<br>- Bacterial pre-peptidase C-terminal domain<br>- Peptidase family M9 N-terminal
PF01757_PF10129	- Acyltransferase family<br>- OpgC protein
PF01797	- Transposase IS200 like
PF01807_PF08275	- CHC2 zinc finger<br>- DNA primase catalytic core N-terminal domain
PF02518_PF13426	- Histidine kinase- DNA gyrase B- and HSP90-like ATPase<br>- PAS domain
PF02668_PF05141	- Taurine catabolism dioxygenase TauD TfdA family<br>- Pyoverdine/dityrosine biosynthesis protein
PF02801	- Beta-ketoacyl synthase C-terminal domain
PF02836_PF02929	- Glycosyl hydrolases family 2 TIM barrel domain<br>- Beta galactosidase small chain
PF02922_PF03714	- Carbohydrate-binding module 48 (Isoamylase N-terminal domain)<br>- Bacterial pullanase-associated domain
PF03099_PF10437	- Biotin/lipoate A/B protein ligase family<br>- Bacterial lipoate protein ligase C-terminus
PF03306	- Alpha-acetolactate decarboxylase
PF03390	- 2-hydroxycarboxylate transporter family
PF04131	- Putative N-acetylmannosamine-6-phosphate epimerase
PF04349	- Periplasmic glucan biosynthesis protein MdoG
PF04393	- Protein of unknown function (DUF535)
PF04434	- SWIM zinc finger
PF04443_PF05893	- Acyl-protein synthetase LuxE<br>- Acyl-CoA reductase (LuxC)
PF04509	- CheC-like family
PF05575	- Vibrio cholerae RfbT protein
PF05946	- Toxin-coregulated pilus subunit TcpA

PF06167	- Phosphoenolpyruvate:glucose-phosphotransferase regulator
PF06340	- Vibrio cholerae toxin co-regulated pilus biosynthesis protein F
PF07055_PF12241	- Enoyl reductase FAD binding domain<br>- Trans-2-enoyl-CoA reductase catalytic region
PF07313	- Protein of unknown function (DUF1460)
PF07459	- CTX phage RstB protein
PF07634_PF11647_PF11713_PF12697	- RtxA repeat<br>- C-terminal region of Pasteurella multocida toxin residues 569-1285<br>- Peptidase C80 family<br>- Alpha/beta hydrolase family
PF07646	- Kelch motif
PF07715	- TonB-dependent Receptor Plug Domain
PF08275_PF08278_PF10410_PF13662	- DNA primase catalytic core N-terminal domain<br>- DNA primase DnaG DnaB-binding<br>- DnaB-helicase binding domain of primase<br>- Toprim domain
PF08765	- Mor transcription activator family
PF09264_PF13088	- Vibrio cholerae sialidase lectin insertion||BNR repeat-like domain
PF09339	- IclR helix-turn-helix domain
PF09900	- Predicted membrane protein (DUF2127)
PF10070	- Uncharacterized protein conserved in bacteria (DUF2309)
PF10671	- Toxin co-regulated pilus biosynthesis protein Q
PF10734	- Protein of unknown function (DUF2523)
PF11612_PF13633	- Type II secretion system (T2SS) protein J<br>- Prokaryotic N-terminal methylation site
PF11661	- Protein of unknown function (DUF2986)
PF11846_PF13425	- Domain of unknown function (DUF3366)<br>- O-antigen ligase like membrane protein
PF12242	- NAD(P)H binding domain of trans-2-enoyl-CoA reductase
PF12614	- Ribosome recycling factor
PF12796	- Ankyrin repeats (3 copies)
PF13172	- PepSY-associated TM helix
PF13570	- YWTD domain
PF13808	- DDE Tnp 1-associated
TIGR02646	- TIGR02646: TIGR02646 family protein
TIGR02675	- tape meas nterm: tape measure domain
Chromosome II	
PF00043_PF13409	- Glutathione S-transferase C-terminal domain<br>- Glutathione S-transferase N-terminal domain
PF00145	- C-5 cytosine-specific DNA methylase
PF00175_PF00970_PF01794	- Oxidoreductase NAD-binding domain<br>- Oxidoreductase FAD-binding domain<br>- Ferric reductase like transmembrane component
PF00589_PF13356	- Phage integrase family<br>- Domain of unknown function (DUF4102)
PF00595_PF03572	- PDZ domain (Also known as DHR or

	GLGF)<br>- Peptidase family S41
PF00768	- D-alanyl-D-alanine carboxypeptidase
PF00984_PF03720_PF03721	- UDP-glucose/GDP-mannose dehydrogenase family central domain<br>- UDP-glucose/GDP-mannose dehydrogenase family UDP binding domain<br>- UDP-glucose/GDP-mannose dehydrogenase family NAD binding domain
PF01035	- 6-O-methylguanine DNA methyltransferase DNA binding domain
PF01381_PF12268	- Helix-turn-helix<br>- Protein of unknown function (DUF3612)
PF01483_PF02128	- Proprotein convertase P-domain<br>- Fungalysin metallopeptidase (M36)
PF01553_PF13444	- Acyltransferase<br>- Acetyltransferase (GNAT) domain
PF01555	- DNA methylase
PF01558_PF01855_PF02775_PF10371_PF12838	- Pyruvate ferredoxin/flavodoxin oxidoreductase<br>- Pyruvate flavodoxin/ferredoxin oxidoreductase thiamine diP-bdg<br>- Thiamine pyrophosphate enzyme C-terminal TPP binding domain<br>- Domain of unknown function||4Fe-4S dicluster domain
PF01609_PF05598	- Transposase DDE domain<br>- Transposase domain (DUF772)
PF01741	- Large-conductance mechanosensitive channel MscL
PF01797	- Transposase IS200 like
PF01966_PF13286	- HD domain<br>- Phosphohydrolase-associated domain
PF02355	- Protein export membrane protein
PF02416	- mttA/Hcf106 family
PF02705	- K+ potassium transporter
PF02810_PF08378	- SEC-C motif<br>- Nuclease-related domain
PF03235	- Protein of unknown function DUF262
PF03441_PF04244	- FAD binding domain of DNA photolyase<br>- Deoxyribodipyrimidine photo-lyase-related protein
PF03693	- Uncharacterised protein family (UPF0156)
PF04151_PF13365	- Bacterial pre-peptidase C-terminal domain<br>- Trypsin-like peptidase domain
PF04365	- Protein of unknown function (DUF497)
PF04970	- Lecithin retinol acyltransferase
PF05015	- Plasmid maintenance system killer protein
PF07081	- Protein of unknown function (DUF1349)
PF07549	- SecD/SecF GG Motif
PF07676	- WD40-like Beta Propeller Repeat
PF07700	- Heme NO binding
PF08173	- Membrane bound YbgT-like protein
PF08668	- HDOD domain
PF08847_PF14280	- Domain of unknown function (DUF1817)<br>- Domain of unknown function (DUF4365)
PF10387	- Protein of unknown function (DUF2442)

PF11185	- Protein of unknown function (DUF2971)
PF11948	- Protein of unknown function (DUF3465)
PF12476_PF13304	- Protein of unknown function (DUF3696)||AAA domain
PF12493	- Protein of unknown function (DUF3709)
PF12686	- Protein of unknown function (DUF3800)
PF13428_PF13519	- Tetratricopeptide repeat<br>- von Willebrand factor type A domain
PF13711	- Domain of unknown function (DUF4160)
PF14355	- Abortive infection C-terminus
TIGR01550	- DOC P1: death-on-curing family protein
TIGR01643	- YD repeat 2x: YD repeat (two copies)
TIGR03660_TIGR03661	- T1SS rpt 143: T1SS-143 repeat domain<br>- T1SS VCA0849: type I secretion C-terminal target domain (VC A0849 subclass)
