# Supplementary figures and images for: Vibrio chromosome-specific families
Source: Front Microbiol. 2014 Mar 18;5:73. doi: 10.3389/fmicb.2014.00073 (PMC3957060; doi:10.3389/fmicb.2014.00073)

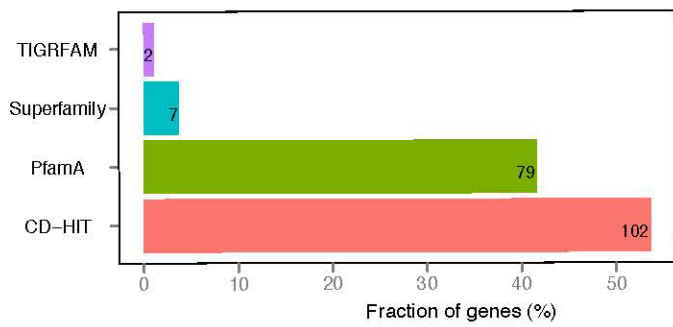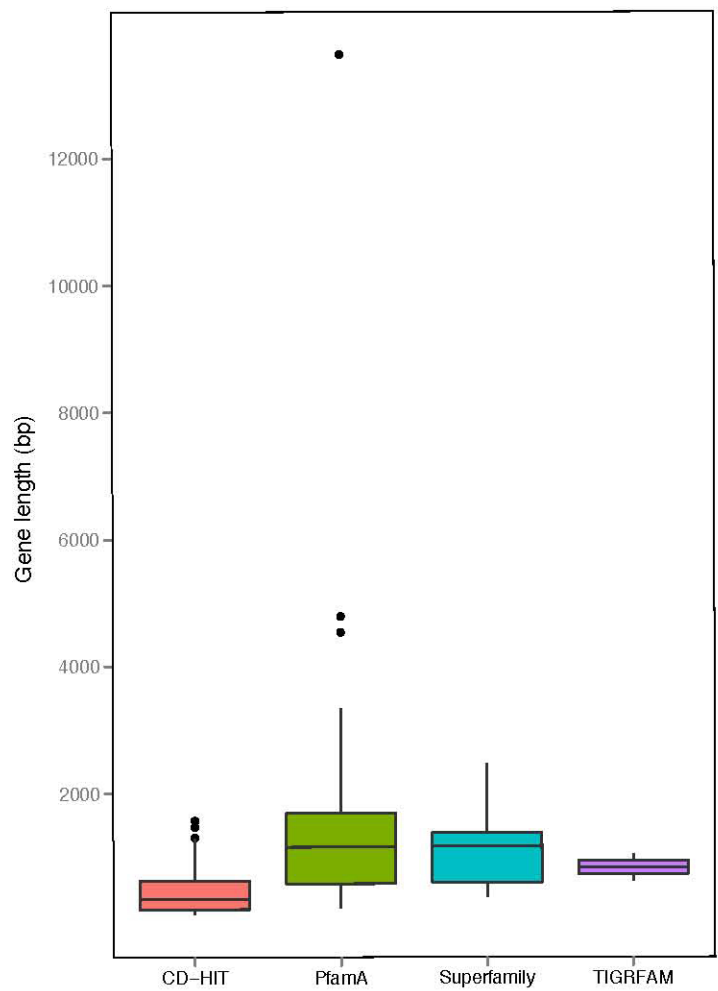

A.

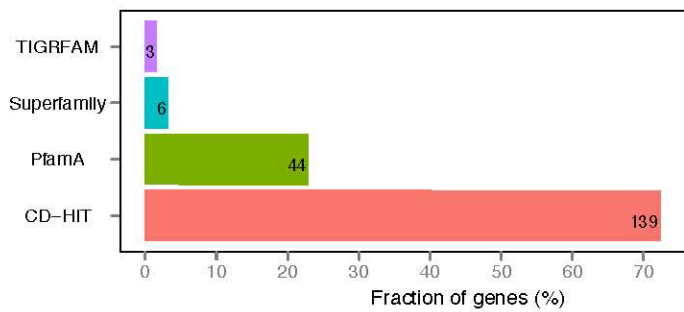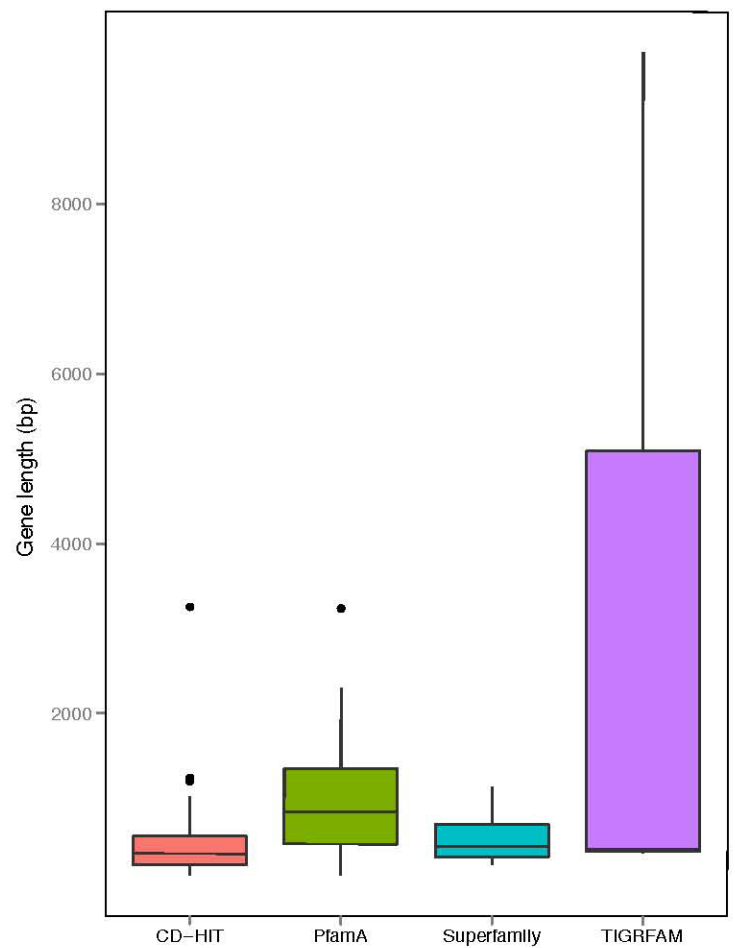

B.

Supplement: Figure S4 — Annotation and length distribution of proteins within V. cholerae species-specific proteomes for chromosome 1 (panel A) and chromosome 2 (panel B). Annotation of profiles and protein coding gene length distribution are visualized by assignment source: PfamA, Superfamily, TIGRFAM, and CD-HIT clustering. [file Presentation1.PDF]
